# Restoration of Ailing Wetlands

**DOI:** 10.1371/journal.pbio.1001248

**Published:** 2012-01-24

**Authors:** Oswald J. Schmitz

**Affiliations:** School of Forestry and Environmental Studies, Yale University, New Haven, Connecticut, United States of America; McGill University, Canada

## Abstract

It is widely held that humankind's destructive tendencies when exploiting natural resources leads to irreparable harm to the environment. Yet, this thinking runs counter to evidence that many ecological systems damaged by severe natural environmental disturbances (e.g., hurricanes) can restore themselves via processes of natural recovery. The emerging field of restoration ecology is capitalizing on the natural restorative tendencies of ecological systems to build a science of repairing the harm inflicted by humans on natural environment. Evidence for this, for example, comes from a new meta-analysis of 124 studies that synthesizes recovery of impacted wetlands worldwide. While it may take up to two human generations to see full recovery, there is promise, given human will, to restore many damaged wetlands worldwide.

## Abstract 2

The science of ecological restoration involves building the technical understanding needed to restore damaged ecosystems, such as wetlands, which provide critical services needed to support human health and economic well-being.

In a now classic essay entitled *Round River* [1], Aldo Leopold—the father of modern environmental ethics—lamented,

“One of the penalties of an ecological education is that one lives alone in a world of wounds … … An ecologist must either harden his shell and make believe that the consequences of science are none of his business, or he must be the doctor who sees the marks of death in a community that believes itself well and does not want to be told otherwise.”

Leopold used the metaphor of integrated medical science and practice to encourage a parallel integrated environmental science and practice in which one studied ecological processes in part to provide the means and capacity to diagnose the environment's ailments and then restore it back to health [2]. His prescience however tended to be lost on generations of ecological scientists and conservation practitioners who instead viewed the world rather dichotomously. There was the built environment where humans went about living; and then there was wild Nature where ecological science could undertake detailed analysis of the processes that shape the diversity of life and associated ecosystem functioning [3]. The business of doing ecological science became tantamount to finding cures for sickness by studying only healthy subjects [3]. The business of conservation practice became one of diagnosing and chronicling human-caused environmental destruction with the intent to spur the protection of Nature within preserves and protected areas that eschewed human presence. This reinforced an approach in ecology and conservation of forecasting perpetual gloom and doom by giving the impression that all human–environmental interactions necessarily lead to irreversible damages [4],[5].

This is not to suggest that human-caused damages to the environment are not prevalent or problematic. Indeed, an expanding human population has translated into increased demand for natural resources and environmental services [4]–[6]. The global human footprint is now so large and far-reaching that some have even begun to question the whole notion that the planet could somehow be rationally divided into places that were available to be domesticated by humans and places that safely remained wild [5]. The reality is that as the biotic and biophysical conditions of the environment become degraded in places where humans exist, they often tend to abandon those places and search for new ones to exploit. But, living on a finite planet with finite space and finite resources means that there is limited if any recourse to continue to abandon degraded areas and shift exploitation to nondegraded ones [5]–[7]. The time has come to operationalize Leopold's vision of an integrated environmental science and practice that provides the scientific understanding and means to restore degraded environments back to health.

That vision, embodied in the idea of “restoration ecology,” connects basic ecological research with the mission to develop techniques for rehabilitating the environment by encouraging natural processes or by translating scientific insights into management to speed up the processes [2],[4],[8]. In some respects, this is a logical outgrowth of classic scientific understanding of the way ecosystems have assembled themselves over time. Ecosystems throughout the globe originated from natural development processes of primary succession or natural restorative processes of secondary succession [4],[9]. Primary succession follows when biotic components of ecosystems become established on barren substrates like lava flows or glacial remains and then build up to form a complex ecosystem. Secondary succession arises on substrates previously occupied by biotic species after major disturbances like fires and floods denuded the areas of the biota. Widespread evidence of ecological succession shows the power of natural processes to re-create ecosystems without help [4]. These principles now form the basis of a new framework for systematic study and reconstruction of ecosystems. The goal of restoration ecology is to raise and answer questions through synthetic analysis of the restorative process [3]. The application of the science involves harnessing this natural capacity by introducing interventions that reverse the effects of long-term problems and steer ecological systems back to their original, natural state. The promise of restoration ecology is that it can create a tool kit of management options to balance environmental protection and providing environmental services for a burgeoning human population.

Realizing the promise, however, requires addressing two looming issues that pose important scientific challenges. First, the idea of designing restoration to emulate or enhance successional processes implies that ecosystems can recover gradually from disturbances [7],[10]. But, there is the potential that disturbances could cause ecosystems to reach critical thresholds causing catastrophic shifts in their state (Figure 1). Restoration efforts may then become a proverbial difficult up-hill climb, if they can be restored at all [7],[10]. Second, ecological science has not been conducted for a sufficient length of time to be able to catalogue what the natural states of the myriad ecological systems of the globe in fact are. Thus, to transfer ecological science into practice, ecologists must first wrestle with defining what it means for an ecosystem to be fully recovered and then, through synthesis of ecological studies, identify conditions likely to lead to full recovery [2],[4],[7],[9],[11],[12].

**Figure 1:**
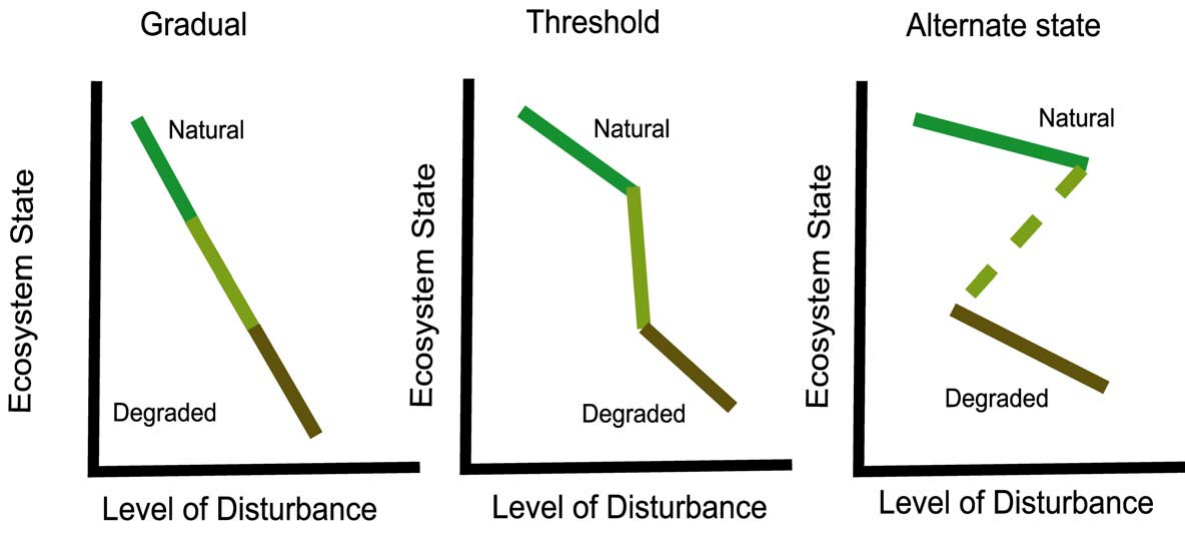
Different potential ways that ecosystem state may change in relation to the level of environmental disturbance. Solid lines denote pathways of state changes from natural to degraded conditions, and the dashed line indicates a transition where the system jumps from a natural to a degraded state. The figure illustrates three general scenarios. Ecosystems may undergo gradual degradation with a rise in disturbance level and may recovery gradually as the disturbance is abated. Ecosystems may exhibit threshold-like behavior in which a certain level of disturbance causes an abrupt change in state and disturbance abatement causes an abrupt “up-hill” return in ecosystem state. Finally, an ecosystem may exhibit a threshold shift in ecosystem state that may only be recoverable with a large turnaround of the critical environmental parameter or disturbance that caused the system to shift from the original state. The scientific challenges in restoration ecology are: characterizing what a natural “green” state is; identifying how long a perturbation must be in place to determine whether the system changes gradually or abruptly; and how long it will take to reverse the effects of a disturbance. Figure is adapted from [10].

A case in point concerns the global need to restore wetlands such as marshes, peatlands, floodplains, mangroves, and brackish estuaries [9]. Relative to their low representation globally (1.5% of the Earth's surface), wetlands provide huge services to humans, valued in multiple trillions of dollars [9]. These highly important ecosystems have, however, suffered some of the greatest levels of destruction of all ecosystem types [13]. These facts necessarily make wetlands important candidates for restoration efforts. But, their successful recovery may be highly contingent upon the landscape context, including surrounding habitat type and land development, hydrological regime and topography, nutrient inputs, and natural disturbance regimes [9]. In this issue of *PLoS Biology*, Moreno-Mateos et al. [14] report on a synthesis of how landscape context influences wetland restoration success in order to establish a scientific prognosis for their recovery.

Moreno-Mateos et al. [14] conducted an exhaustive search of the scientific literature and identified about 3,000 studies that report on wetland restoration efforts. They then filtered this list of studies using stringent criteria needed to judge restoration success. Foremost, the study had to have an undisturbed reference to serve as a natural state against which to compare the degree of wetland damage and recovery. The study also had to focus on natural wetlands, as opposed to highly engineered artificial systems. Finally, the study had to be conducted over long time periods in order to determine if the wetland is undergoing either gradual recovery, threshold-like recovery, or is locked in an alternative state. These criteria were met in only 4% of the approximately 3,000 studies. Granted, that 4% (124 studies) is a sufficient number to undertake a scientifically defensible synthetic analysis; but, this limitation in the number of rigorous studies faced here, with similar constraints faced by other syntheses of ecological restoration [7],[12], highlights that the science of restoration ecology is still very much in its infancy in its ability to gauge restoration successes.

The synthesis of the 124 wetland restoration studies revealed that recovery of the physical and biotic properties and the functioning of wetland ecosystems proceeded on different time scales. Active restoration of wetland physical features like topography, soil permeability, surface and ground water flows lead to immediate recovery. The abundance and composition of wetland vertebrate species recovered to reference levels usually within 5 years. Large aquatic invertebrates took 5 to 10 years to approach reference levels, but in many cases did not reach absolute reference levels. Plant assemblages took on average 30 years to converge on reference states. Finally, it took 50 to 100 years for wetlands to recover normal nutrient cycling. Interestingly, these time scales are on par with the time course of secondary succession following natural disturbances [7]. Consistent with expectations for wetland systems [9], the rate of recovery varied with the environmental context. Larger wetlands recovered more quickly than smaller wetlands. Wetlands in warmer climates recovered more rapidly than in colder climates. Wetlands connected to other wetlands via intact hydrological structure tended to recover more rapidly than isolated wetlands. All told, in most cases the systems tended to recover rather than be locked in an alternate state.

In an ideal world, society would exploit ecological systems in ways that ensure long-term sustainability of their structure and function rather than degrade them. But, with even the best sustainable practices in place, unforeseen outcomes and damages can happen accidentally [4],[5]. Moreno-Mateos et al.'s study provides evidence that given human will, it is possible to restore human-damaged ecosystems on timescales of one to two human generations. On a societal level, the promise of restoration ecology demonstrated in this synthesis and other recent syntheses [7],[12] first helps to dispel the notion that human activity necessarily has irreversible negative impacts on ecosystems [3],[4],[7] and, second, shows that with ecological know-how and application, it is possible to cure some ailing environments.
